# Environmental Stress Induces Altered Composition of *Streptococcus mutans* Membrane Vesicles: pH‐Driven Changes in Membrane Vesicle Production and Composition

**DOI:** 10.1111/omi.70022

**Published:** 2026-02-20

**Authors:** Taylor C. Boone, Swetha K. Shankar, Melodie L. Weller

**Affiliations:** ^1^ Department of Pathology, Division of Microbiology and Immunology University of Utah Salt Lake City Utah USA; ^2^ School of Dentistry University of Utah Salt Lake City Utah USA

**Keywords:** bacterial membrane vesicles (MVs), environmental stress, gram‐positive bacteria, nanoparticles, pH, *Streptococcus mutans*

## Abstract

Bacteria produce membrane vesicles (MVs) in response to environmental stress and genetic changes. Previous studies have shown that MVs can trigger inflammatory responses and may serve as important mediators of host–microbe interactions. Given the dynamic nature of the oral microbiome, bacteria such as *Streptococcus mutans* are frequently exposed to environmental fluctuations that could alter MV production. The objective of this study was to investigate whether inducing stress conditions would affect MV production and morphology in *S. mutans*, a prominent oral pathogen. Cultures were subjected to different pH conditions to mimic environmentally relevant stress. MVs were isolated and purified in order to characterize and assess changes in yield, size, and cargo. Our findings show that acidic stress significantly increased MV production while reducing average MV size. We also observed significant differences in MV content when compared to control conditions. These changes may reflect bacterial adaptation strategies and could influence how MVs interact with host immune systems. Overall, this study highlights the potential for environmental stress to reshape MV‐mediated communication in the oral microbiome and provides a foundation for exploring how such changes may contribute to inflammation and oral disease.

## 1 Introduction

**FIGURE 1:**
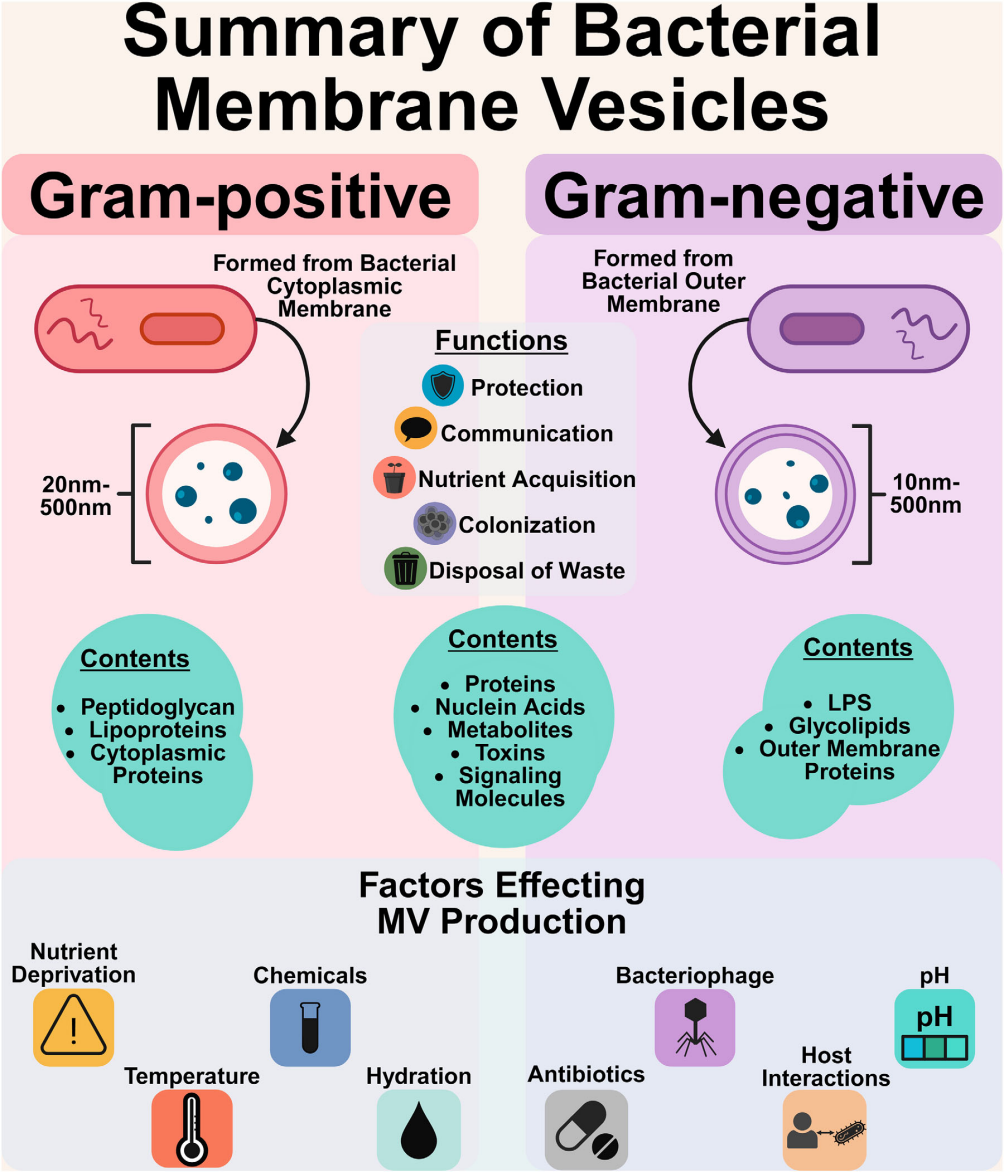
Summary of bacterial membrane vesicles. Environmental factors such as pH, temperature, hydration, nutrient deprivation, bacteriophage, chemicals, host interactions, and antibiotics further regulate MV production, shaping their role in disease progression (Mozaheb and Mingeot‐Leclercq 2020). Gram‐positive bacterial membrane vesicles (MVs) originate from the cytoplasmic membrane, while Gram‐negative membrane vesicles (OMVs) are derived from the outer membrane. Both types of MVs play essential roles in protection, communication, and colonization. They contain a diverse array of proteins, nucleic acids, and toxins. Gram‐positive MVs are enriched with peptidoglycan, lipoproteins, and cytoplasmic proteins, whereas Gram‐negative MVs contain lipopolysaccharides (LPS), glycolipids, and outer membrane proteins (Gill et al. 2019). MV production is influenced by various factors, including nutrient availability, pH, and bacteriophage interactions.

Membrane vesicles (MVs) contribute to bacterial processes such as immune evasion, nutrient acquisition, and progression of disease (Figure 1) (Mozaheb and Mingeot‐Leclercq 2020; Wu et al. 2022). MVs are released by many cell types, including both prokaryotic and eukaryotic cells, and are formed by budding off the cell surface (Figure 1) (Gill et al. 2019; Schorey et al. 2021). In addition to roles in communication and adaptation, bacterial MVs contribute to host–pathogen interactions, influencing immune responses and disease outcomes across multiple organ systems (Gill et al. 2019; Mozaheb and Mingeot‐Leclercq 2020). MVs play key roles in conditions such as airway inflammation (Ryu et al. 2023), sepsis (Tian et al. 2023), and periodontal disease (S. Chen et al. 2023). Bacterial MVs, produced by both Gram‐positive and Gram‐negative bacteria, contribute to disease by modulating immune responses, delivering toxins, and upregulating proinflammatory cytokines, contributing to the onset and severity of these conditions (Figure 1) (S. Chen et al. 2023).

Bacterial MVs vary in size and composition by species, with sizes ranging from 10 to 1000 nm (Bose et al. 2020; Gill et al. 2019; Rutter and Innes 2023). Both pathogenic and nonpathogenic organisms release MVs (Figure 1) (Deatherage and Cookson 2012). Bacterial MVs, including outer‐membrane vesicles (OMVs) from Gram‐negative bacteria, typically range from 10 to 500 nm, while those from Gram‐positive bacteria range from 20 to 500 nm (Figure 1) (Bose et al. 2020; Xu et al. 2023). OMVs were first described in 1965 in a strain of *Escherichia coli*, where they were observed to release high levels of lipopolysaccharides (Bishop and Work 1965). In contrast, Gram‐positive cells have a thicker peptidoglycan layer than Gram‐negative cells, which influences MV biogenesis (Figure 1) (Liu et al. 2018; Villageliu and Samuelson 2022). Bacterial MVs carry proteins, nucleic acids, toxins, and other molecules from their parent cells, enabling diverse functions such as immune evasion, communication, and environmental adaptation (Figure 1) (Gill et al. 2019; Mozaheb and Mingeot‐Leclercq 2020). Environmental factors such as pH and temperature further regulate MV production, shaping their role in disease progression (Figure 1) (Mozaheb and Mingeot‐Leclercq 2020).

The purpose of MVs varies between different types of bacteria. There are several functions shared across MVs, including acting as decoys for bacteriophages and antibiotics, aiding in nutrient acquisition, and facilitating waste disposal (Figure 1) (MacNair and Tan 2023; Mozaheb and Mingeot‐Leclercq 2020; Reyes‐Robles et al. 2018; Xiu et al. 2024). Other MV functions may be species specific, such as transferring DNA or other cargo and expression of toxins (Gill et al. 2019).

Among the many bacteria that produce MVs, *Streptococcus mutans*, a Gram‐positive bacteria found in the human oral cavity, is notable for its role in forming dental plaque (biofilm) on the hard surface of teeth (Lemos et al. 2019; Matsumoto‐Nakano 2018). This bacteria is well‐suited to thrive in harsh environments, such as low pH conditions, due to its robust acid tolerance response (Baker et al. 2017). This response allows *S. mutans* to maintain an internal pH that is more alkaline than its acidic surroundings, protecting its cellular machinery from damage (Baker et al. 2017). Additionally, the bacteria adapts by altering the composition of its membrane fatty acids, which reduces proton permeability and prevents excessive acidification inside the cell (Baker et al. 2017). These adaptations collectively enable *S. mutans* to withstand acidic stress, making it a primary contributor to dental caries (Baker et al. 2017). *Streptococcus mutans* MVs were first identified in 2014 (Liao et al. 2014). Like other bacteria, *S. mutans* MVs participate in processes such as intercellular signaling, biofilm development, and disease progression (Wu et al. 2022). Previous studies have characterized the proteomic and metabolomic profile of *S. mutans* MVs produced at different pH values (7.5 vs. 5.5), showing that acidic conditions increased MV release, reduced vesicle size, and altered vesicle‐associated proteins and metabolites (Cao et al. 2020). However, that study did not investigate the RNA cargo of MVs, leaving open how environmental pH affects RNA packaging into vesicles.

While bacterial MVs play crucial roles in host interactions and disease progression, their specific functions can vary across bacterial species. Given the crucial role of the oral microbiome in health and disease (Deo and Deshmukh 2019), this study investigated how environmental shifts, specifically changes in pH, influence the production and properties of *S. mutans* MVs.

## 2 Methods

### 2.1 Bacterial Culture Conditions

*Streptococcus mutans* UA159 (ATCC, strain NCTC 10449) was initially cultured overnight in 30 mL of brain heart infusion (BHI) broth (BD Difco) at 37°C under aerobic conditions with agitation to avoid sedimentation. This approach is commonly used in *S. mutans* physiological and stress‐response studies to promote uniform aeration (Ahn et al. 2007). The overnight culture was subsequently diluted 1:100 into fresh BHI broth supplemented with 1% (w/v) sucrose to promote biofilm‐associated growth. Two experimental conditions were established by adjusting the medium to either neutral pH (7.2) or acidic pH (5.2) using sterile 1 M HCl or NaOH as required. Cultures were incubated overnight at 37°C in a shaking incubator to ensure aeration and uniform growth prior to downstream analyses. *Streptococcus mutans* UA159 (ATCC, strain NCTC 10449) was used for all experiments. The complete UA159 genome sequence (Ajdić et al. 2002) served as the reference for RNA‐seq alignment.

### 2.2 Growth Curve Analysis

Overnight cultures of *S. mutans* were diluted 1:10 into fresh BHI media and dispensed into 96‐well plates. Plates were sealed with optically clear adhesive film to minimize evaporation and incubated at 37°C in a plate reader (BioTek Synergy HTX, Agilent Technologies). Bacterial growth was monitored by measuring the optical density at 600 nm (OD600) at 30‐min intervals for up to 42 h. Colony‐forming unit (CFU) equivalents were estimated using the equation CFU = OD600 × conversion factor, where the conversion factor for *S. mutans* was previously determined to be approximately 1.3 × 10^8^ CFU/mL (Kim et al. 2013).

### 2.3 MV Isolation and Purification

MVs from *S. mutans* were isolated from liquid cultures as previously described, with modifications (Wang et al. 2018). Briefly, bacterial cells were removed by centrifugation at 3500 × *g* for 15 min at 4°C, and the resulting supernatant was clarified by an additional centrifugation at 10,000 × *g* for 15 min at 4°C. The supernatant was then passed through 0.22‐µm polyethersulfone (PES) filters (MilliporeSigma) to remove residual cell debris. Vesicles were pelleted by ultracentrifugation at 100,000 × *g* for 70 min at 4°C (Beckman Coulter Optima L‐100 XP, Brea, CA, USA) and resuspended in phosphate‐buffered saline (PBS). Crude MV preparations were stored at −80°C until further processing. A mixture of centrifugation and filtration is widely used and accepted for bacterial MV studies (Reimer et al. 2021). All downstream analyses, including NTA, protein and RNA quantification, RNA extraction, and RNA sequencing, were performed on MVs that underwent the workflow described above.

**FIGURE 2:**
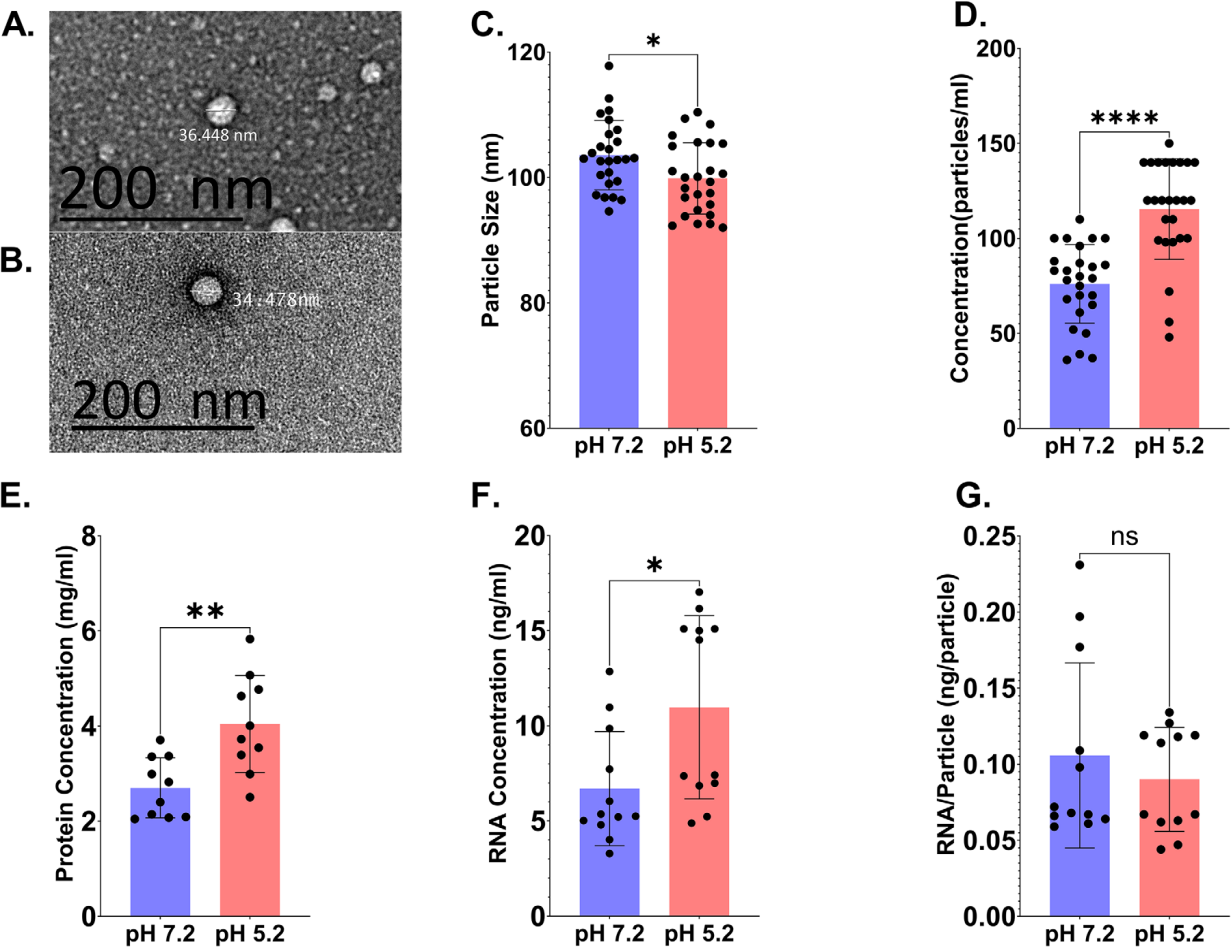
MV profiles altered based on environmental conditions. (A, B) *S. mutans* bacterial membrane vesicles grown at a pH of 7.2 (A) and 5.2 (B) were visualized using negative staining transmission electron microscopy (TEM), revealing their size, shape, and structural integrity. For nanoparticle tracking analysis (NTA), Zetaview was used to assess and compare the size distribution and concentration of isolated particles in both conditions. (C) Average size of MVs using NTA (*N* = 26). (D) Average concentration of MVs using NTA (*N* = 26). (E) Protein concentrations measured in vesicles from each growth condition (*n* = 10). (F) RNA concentrations measured in vesicles grown at each pH level, highlighting any differences in packaging between the two conditions (*n* = 12). (G) The amount of RNA in each individual membrane vesicle particle (*n* = 12). *p < 0.05; **p < 0.01; ****p < 0.0001.

For additional purification, a density gradient was used. MVs were layered on OptiPrep (iodixanol) gradients prepared in PBS as previously described (Wang et al. 2018). Briefly, 1 mL of crude MV suspension was mixed with 1 mL of 60% OptiPrep and overlaid sequentially with 2 mL each of 30%, 25%, 20%, 15%, and 10% OptiPrep dilutions. Gradients were centrifuged at 140,000 × *g* for 19 h at 4°C, after which twelve 1‐mL fractions were collected from the top. Fractions were analyzed by SDS‐PAGE with silver staining, and vesicle‐containing fractions were pooled and further characterized by transmission electron microscopy (TEM). Only samples from Figure 2A,B underwent density gradient purification.

### 2.4 TEM

Negative‐stain TEM was used to visualize isolated and density‐gradient–purified MV preparations. Briefly, 5 µL of the sample was applied to a copper grid (Electron Microscopy Sciences) and allowed to adsorb for 3 min. Excess liquid was gently blotted with filter paper, and grids were rinsed briefly in sterile distilled water for 3 s before blotting again. Grids were then stained with 5.5 µL of uranyl acetate for 30 s, blotted to remove excess stain, and air‐dried at room temperature prior to imaging. Samples were examined using a JEOL JEM‐1400‐PLUS. Processing and imaging were performed by the University of Utah EM Core.

### 2.5 Nanoparticle Tracking Analysis (NTA)

Quantification and size distribution of *S. mutans* MVs were assessed using a ZetaView Nanoparticle Tracking Analyzer (Particle Metrix) equipped with 488‐, 520‐, and 640‐nm lasers. Vesicle suspensions were diluted in sterile 1× PBS to achieve a particle concentration within the optimal range of 1 × 10^7^ to 1 × 10^9^ particles/mL. Data acquisition and analysis were performed using the ZetaView software package (version 8.06.01, Particle Metrix).

### 2.6 RNA Isolation and Sequencing

Small RNAs were isolated from MV preparations using the PureLink miRNA Isolation Kit (Thermo Fisher Scientific) according to the manufacturer's protocol. Although marketed for miRNA isolation, this kit extracts a broad range of small RNAs, including tRNAs, tRNA fragments (tRFs), sRNAs, and rRNA fragments, which is appropriate for MV‐associated RNA because vesicles are enriched in smaller RNA classes. To enable comparison between MVs and whole‐cell RNA profiles, small RNA from whole cells was isolated using the same protocols. RNA concentration and quality were assessed using the Qubit RNA High Sensitivity Assay Kit (Thermo Fisher Scientific) on a Qubit 4 Fluorometer. RNA quality was additionally assessed using an Agilent Bioanalyzer, and libraries were prepared at the University of Utah High‐Throughput Genomics (HTG) core using the QIAseq miRNA Library Kit (UDI; Qiagen). Libraries were sequenced on an Illumina NovaSeq platform to generate paired‐end 150‐bp FASTQ files. Adapter trimming and quality filtering were performed using CLC Genomics Workbench 22.0.2. For *S. mutans* whole‐cell transcriptome, total RNA was isolated using Qiagen RNeasy kit with Trizol pretreatment (Speer and Poole 2018). Full *S. mutans* transcriptomic library preparation and sequencing were performed by SeqCenter, LLC (Pittsburgh, PA, USA). Reads were aligned to the *S. mutans* UA159 reference genome (Ajdić et al. 2002). RNA‐seq analysis was employed following the manufacturer's instructions to generate miRNA sequencing libraries. Libraries were then sequenced at the HTG core at the University of Utah.

### 2.7 RNA‐seq and Pathway Analysis

RNA‐seq analyses were performed using CLC Genomics Workbench (v22.0.2). Differentially expressed genes were identified and filtered based on a false discovery rate (FDR) *p*‐value threshold of <0.05. Pathway enrichment for the *S. mutans* whole‐cell transcriptome was further analyzed for pathway enrichment using the Biocyc database, and the significance of pathway enrichment or depletion was assessed using *p*‐values calculated by the Biocyc platform (BioCyc Pathway/Genome Database Collection [https://biocyc.org/]).

### 2.8 Protein Extraction and Analysis

Proteins were solubilized by the addition of NP‐40 to each sample. Protein concentrations were determined using the DC Protein Assay Kit II (Bio‐Rad Laboratories) following the manufacturer's instructions, and absorbance was measured at 750 nm using a plate reader (BioTek Synergy HTX, Agilent Technologies). For SDS‐PAGE, equal amounts of protein (normalized by concentration) were resolved using MES buffer (Thermo Fisher Scientific). Gels were subsequently stained using the Pierce Silver Stain Kit (Thermo Fisher Scientific). Band sizes were estimated using ImageJ.

### 2.9 Statistical Analysis

Statistical analyses were performed using GraphPad Prism, Biocyc, and the CLC Genomics Workbench software. Data were assessed for distribution, and comparisons between pH conditions were carried out using unpaired *t*‐tests for normally distributed datasets.

### 2.10 Data Availability

Sequencing datasets have been deposited into the NCBI database as PRJNA1413374.

## 3 Results

### 3.1 Morphological and Biochemical Characterization of *S. mutans* MVs at Different pH Conditions Reveals Distinct MV Properties

*Streptococcus mutans* was cultured under different pH conditions to study how environmental changes affect the characteristics of its MVs. Growth curves for cultures initiated at pH 7.2 and pH 5.2 are shown in Figure S1, confirming that cultures grown at pH 5.2 exhibited slower early growth compared to those started at pH 7.2, consistent with established effects of acidic stress on *S. mutans* physiology. Negative staining TEM was used to visualize MVs produced at pH 7.2 and pH 5.2 in order to determine MV size and morphology (Figure 2A,B). NTA was performed to provide additional insights into MV size and concentration. The analysis showed a decrease in MV size at pH 5.2 (103.6 ± 5.545 for pH 7.2 and 99.86 ± 5.697 for pH 5.2; Figure 2C). However, the concentration of MVs produced under acidic conditions exhibited an increase, indicating potential alterations in MV production or stability at lower pH levels (76.08 ± 20.65 for pH 7.2 and 115.4 ± 26.49 for pH 5.2; Figure 2D).

To further investigate the biochemical properties of these MVs, protein concentrations were measured. A significant increase in protein levels was observed in MVs grown at pH 5.2 compared to those grown at pH 7.2, suggesting altered protein packaging under acidic conditions (2.701 ± 0.6311 for pH 7.2 and 4.046 ± 1.02 for pH 5.2; Figure 2E). MVs grown at pH 7.2 and pH 5.2 exhibited distinct protein packaging profiles, with three key proteins at approximately 160, 67, and 61 kDa showing differential packaging between the two conditions (Figure S2). Although the silver‐stained gel in Figure S2 appears to show more intense bands for the pH 7.2 sample, each lane contained an equal amount of protein (20 µg), as determined by using a Bradford Assay. These findings indicate that acidic stress may influence not only MV production but also protein content, potentially altering MV‐mediated interactions with host cells.

RNA concentrations were also assessed, revealing an increase in RNA content in MVs grown at pH 5.2 (6.701 ± 2.999 for pH 7.2 and 10.97 ± 4.814 for pH 5.2; Figure 2F). Interestingly, despite the increased production of MVs under stressed conditions and their smaller size, the RNA content per individual MV remained unchanged (0.1058 ± 0.06086 for pH 7.2 and 0.09008 ± 0.03422 for pH 5.2; Figure 2G). This suggests that while stress influences MV quantity and size, it does not significantly affect the RNA concentration per vesicle. Together, these results highlight a complex relationship between environmental pH and MV biogenesis, suggesting that acidic conditions may enhance MV biogenesis, affecting both vesicle size and cargo content.

### 3.2 The RNA Composition of *S. mutans* MVs Changes Under Different pH Conditions

**FIGURE 3:**
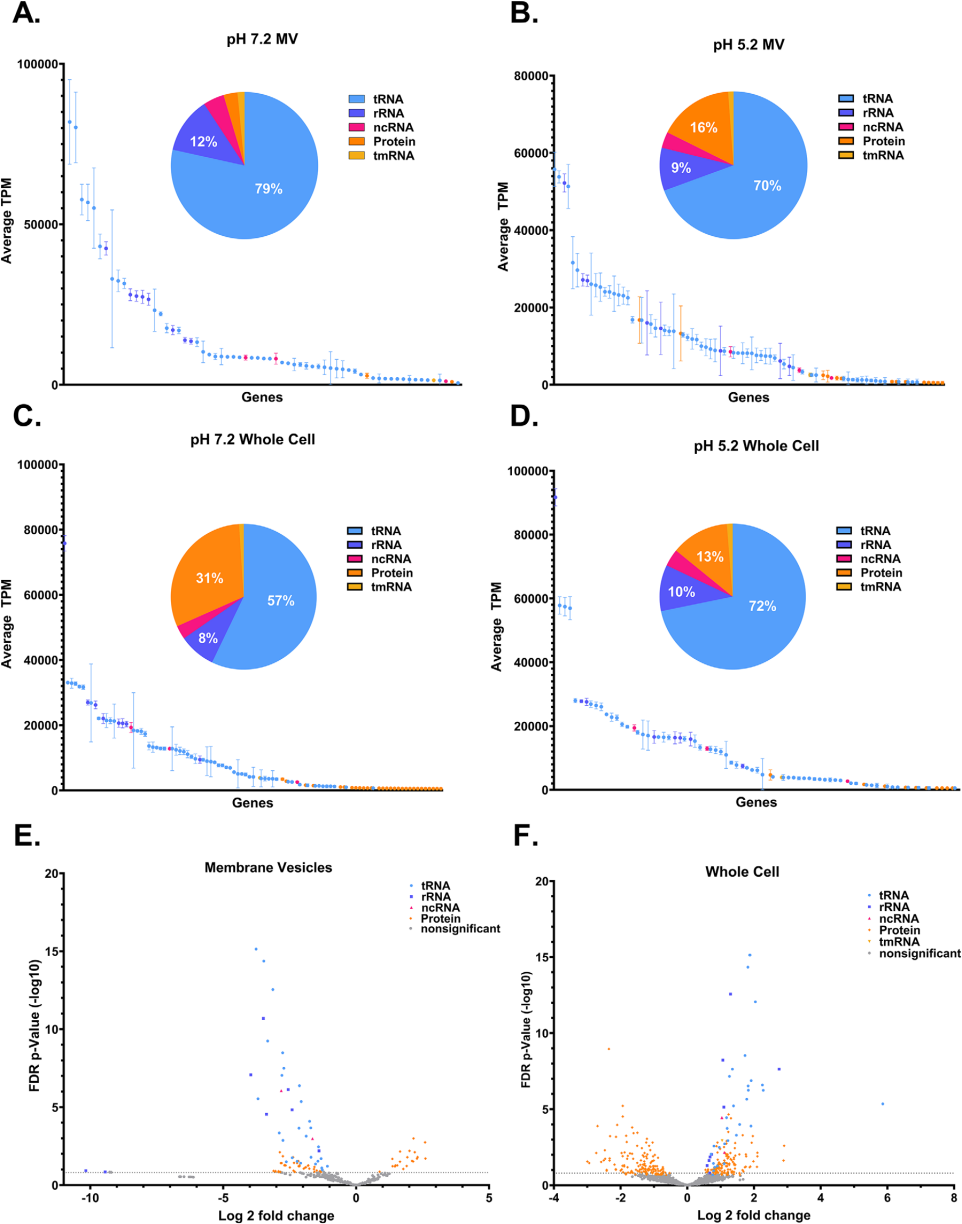
Differential RNA profiles in MVs and *S. mutans* whole cells as a function of environmental conditions. Transcriptomic analysis of *S. mutans* MVs. (A) RNA packaging patterns in MVs grown at pH 7.2, including average TPM values (above 500 TPM), the most abundant transcripts, and their proportional distribution in a pie chart. (B) RNA packaging patterns in MVs grown at pH 5.2, including average TPM values (above 500 TPM), the most abundant transcripts, and their proportional distribution in a pie chart. (C) RNA packaging patterns in small RNA within the whole cells grown at pH 7.2, including average TPM values (above 500 TPM), the most abundant transcripts, and their proportional distribution in a pie chart. (D) RNA packaging patterns in small RNA within the whole cells grown at pH 5.2, including average TPM values (above 500 TPM), the most abundant transcripts, and their proportional distribution in a pie chart. (E) Volcano plot from MVs, displaying transcripts that are differentially expressed between pH 7.2 and pH 5.2, illustrating upregulated and downregulated transcripts. (F) Volcano plot from small RNA within the whole cells, displaying transcripts that are differentially expressed between pH 7.2 and pH 5.2, illustrating upregulated and downregulated transcripts.

RNA sequencing was performed on MVs as well as whole‐cell *S. mutans* under both pH 7.2 and pH 5.2 culture conditions to examine differences in RNA composition (Figure 3). Figure 3A highlights RNAs with transcript levels above 500 transcripts per million (TPM) in MVs produced at pH 7.2. Notably, about 79% of the identified transcripts were associated with tRNA, 12% with rRNA, 5% with ncRNA, 3% with protein‐coding RNA, and 2% with transfer‐messenger RNA (tmRNA). For MVs produced at pH 5.2, Figure 3B shows RNAs exceeding 500 TPM, with specific categories including about 70% tRNA, 9% rRNA, 4% ncRNA, 16% protein‐coding RNA, and 1% tmRNA. The observed shift in RNA distribution under acidic conditions suggests that environmental stress may influence the selective packaging of RNA classes into MVs.

Whole‐cell RNA‐seq was analyzed in parallel with MV RNA‐seq under identical growth conditions (pH 7.2 and pH 5.2) to directly compare cellular and vesicle‐associated RNA profiles. This comparison revealed several differences in RNA composition between the two conditions and between the whole cell and MVs. Notably, the small RNA distribution of *S. mutans* grown at pH 7.2 differed in comparison to the MVs grown at the same growth condition. Among small RNA classes with a greater abundance than 500 TPM, RNA isolated from whole‐cell *S. mutans* grown at pH 7.2 contained 57% tRNA, 8% rRNA, 3% ncRNA, 31% protein‐coding RNAs, and 1% tmRNA (Figure 3C). In contrast, whole‐cell small RNA from *S. mutans* grown at pH 5.2 consisted of 72% tRNA, 10% rRNA, 4% ncRNA, 13% protein‐coding RNA, and 1% tmRNA (Figure 3D).

A comparative analysis of RNA‐seq data was conducted to assess differences in small RNA abundance between MVs produced at pH 7.2 and pH 5.2 by examining fold change across RNA classes (Figure 3E,F). Only RNAs exhibiting statistically significant differential abundance (*p* < 0.05) were included in this analysis. This analysis revealed pH‐dependent shifts in MV‐associated RNA composition. MVs produced at pH 5.2 exhibited predominantly negative fold changes in tRNA, rRNA, and ncRNA, indicating reduced representation of these RNA classes under acidic conditions, whereas protein‐coding RNAs displayed both positive and negative fold changes (Figure 3E). In contrast, whole cells of *S. mutans* showed an opposite trend, with tRNA, rRNA, ncRNA, and tmRNA mainly exhibiting positive fold changes, while protein‐coding RNAs again displayed both positive and negative fold changes (Figure 3F). Together, these opposing patterns suggest that acidic conditions alter RNA distribution between whole cells and MVs, consistent with differential RNA distribution during MV formation.

### 3.3 Differential RNA Representation and Genomic Distribution of RNA in *S. mutans* MVs at Different pH Conditions Suggest That Packaging of RNA Into MVs Is pH‐Dependent and May Influence Functionality

**FIGURE 4:**
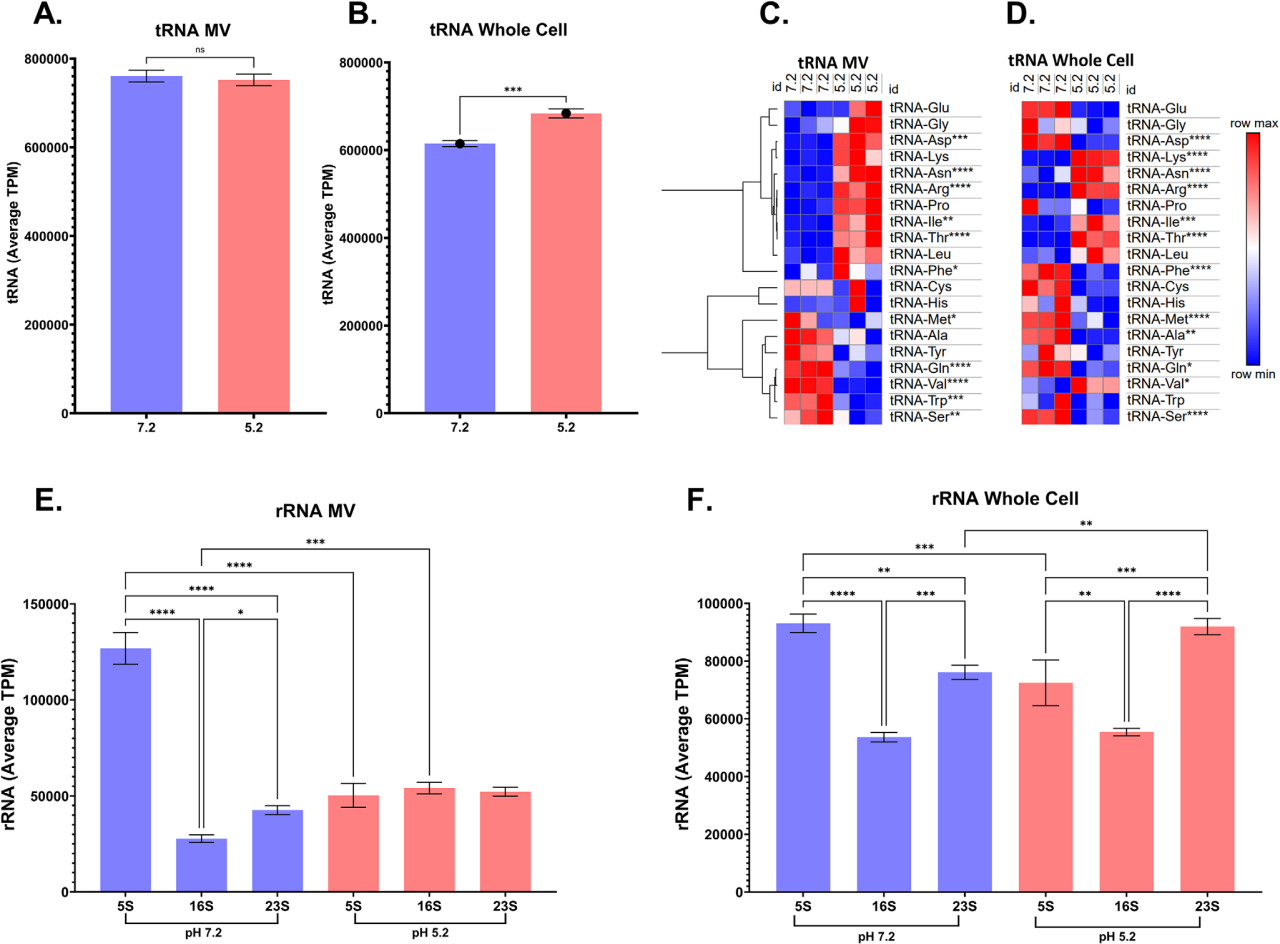
pH‐dependent shift in RNA cargo in MVs. Comparative analysis of RNA‐seq data from *S. mutans* membrane vesicles (MVs) and whole cells grown at pH 7.2 and pH 5.2, highlighting differential RNA packaging between the two conditions. (A) The average TPM of tRNA in both growth conditions from the MVs. (B) The average TPM of tRNA in both growth conditions from small RNA within the whole cells. (C) Different types of tRNAs identified within the MVs, with significant differences indicated by *p < 0.05, **p < 0.01, or ****p < 0.0001. (D) Different types of tRNAs identified within the small RNA in the whole cells, with significant differences indicated by *p < 0.05, **p < 0.01, or ****p < 0.0001. (E) Most common types of rRNA detected in MVs, with significant differences indicated by ***p < 0.001 or ****p < 0.0001. (F) Most common types of rRNA detected in small RNA within the whole cells, with significant differences indicated by ***p < 0.001 or ****p < 0.0001. *n* = 3.

Analysis of tRNA abundance in MV RNA‐seq revealed no significant overall differences in total tRNA levels between the two growth conditions overall (760,844 ± 13,611 for pH 7.2 and 752,211 ± 13,302 for pH 5.2; Figure 4A). In contrast, whole‐cell *S. mutans* RNA‐seq showed a significant increase in tRNA abundance at pH 5.2 compared to pH 7.2 (614,837 ± 6742 for pH 7.2 and 683,885 ± 10,486 for pH 5.2; Figure 4B). However, upon examining individual tRNAs, we observed differences in individual aminoacyl‐tRNAs (Figures 4C,D and S3). Significant variation was observed among individual tRNA types across growth conditions (Figures 4C,D and S3).

For instance, tRNA‐Arg was notably more abundant in MVs and whole cells derived from cultures grown at pH 5.2, while tRNA‐Ser was significantly more enriched in MVs and whole cells grown at pH 7.2 (Figures 4C,D and S3). Notably, several tRNAs, like tRNA‐Glu, tRNA‐Gly, tRNA‐Asp, tRNA‐Pro, tRNA‐Phe, and tRNA‐Val, displayed opposing abundance trends between pH 7.2 and pH 5.2 in whole cells and MVs (Figure 4C,D). For instance, tRNA‐Glu was enriched in MVs at pH 5.2 but depleted in whole cells under the same condition, whereas tRNA‐Val exhibited reduced abundance in MVs but increased abundance in whole cells at pH 5.2. Together, these findings indicate that changes in individual tRNA representation within MVs do not uniformly reflect whole‐cell tRNA abundance and are consistent with condition‐dependent differences in tRNA distribution between the cell and the MV.

To further assess differences in ribosomal RNA (rRNA) representation, we analyzed the average TPM values of small rRNAs, including 5S, 16S, and 23S rRNAs, in both MVs and whole cells of *S. mutans* under growth conditions at pH 7.2 and pH 5.2 (Figure 4E,F). In MVs, there was a significant increase in 5S rRNA abundance at pH 7.2 compared to pH 5.2, whereas 16S rRNA exhibited significantly higher abundance at pH 5.2 relative to pH 7.2 (Figure 4E). When examining rRNA composition within MVs at pH 7.2, both 5S and 23S rRNA were significantly more abundant than 16S rRNA; however, no significant differences among rRNAs were observed within MVs produced at pH 5.2.

In whole‐cell RNA, significant differences in rRNA abundance were also detected between growth conditions. Specifically, 5S rRNA abundance was significantly elevated at pH 7.2 compared to pH 5.2, while 23S rRNA showed increased abundance at pH 5.2 relative to pH 7.2 (Figure 4F). Comparison of TPM values across both conditions also showed notable variation, emphasizing the differential RNA packaging patterns influenced by the environmental pH. At pH 7.2, whole‐cell rRNA distribution resembled that observed in MVs, whereas at pH 5.2, 23S rRNA was significantly more abundant than both 5S and 16S rRNAs, and 5S rRNA was also significantly enriched relative to 16S rRNA (Figure 4E,F). Together, these findings demonstrate that environmental pH influences rRNA composition in both whole cells and MVs, with distinct patterns observed between the two. The differential representation of rRNA in MVs compared to whole cells suggests that rRNA content within the MVs does not simply reflect cellular rRNA abundance and may be shaped by pH‐dependent stress‐related adaptation mechanisms.

### 3.4 Transcriptomic Analysis of *S. mutans* Shows Differential Gene Expression

To assess how acidic pH reshapes the cellular physiology of *S. mutans*, whole‐cell RNA‐seq differential expression data were analyzed using Biocyc pathway enrichment analysis. This approach identifies metabolic and cellular pathways whose constituents are differentially expressed at pH 5.2 relative to pH 7.2. Pathway enrichment significance was determined using the *p*‐value generated by the Biocyc platform.

**FIGURE 5:**
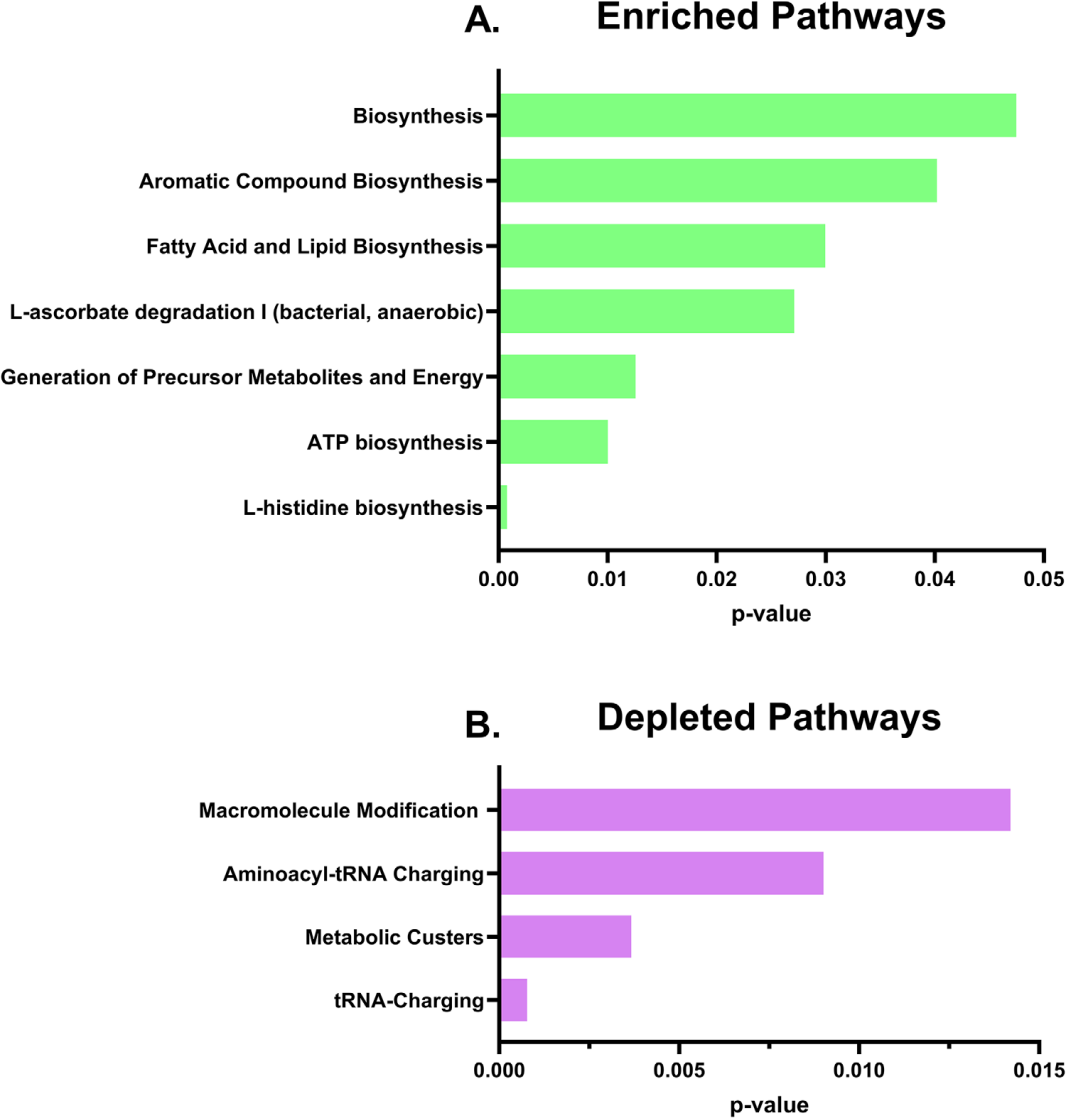
Pathway enrichment analysis of whole‐cell *Streptococcus mutans* RNA‐seq under acidic conditions. Differentially expressed genes identified by whole‐cell RNA‐seq were analyzed using the BioCyc database to identify pathways significantly enriched or depleted under acidic conditions. (A) Pathways that are enriched (Green). (B) Pathways that are depleted (Purple).

Pathways significantly enriched at pH 5.2 included biosynthesis pathways, aromatic compound biosynthesis, fatty acid and lipid biosynthesis, l‐ascorbate degradation I (bacterial, anaerobic), generation of precursor metabolites and energy, ATP biosynthesis, and l‐histidine biosynthesis (Figure 5A). In contrast, several pathways were significantly depleted under acidic conditions, including macromolecule modification, aminoacyl‐tRNA charging, metabolic clusters, and tRNA charging (Figure 5B). Together, these pathway changes indicate a shift in cellular metabolism and translational regulation under acidic stress, consistent with increased energetic demands and MV formation.

**FIGURE 6:**
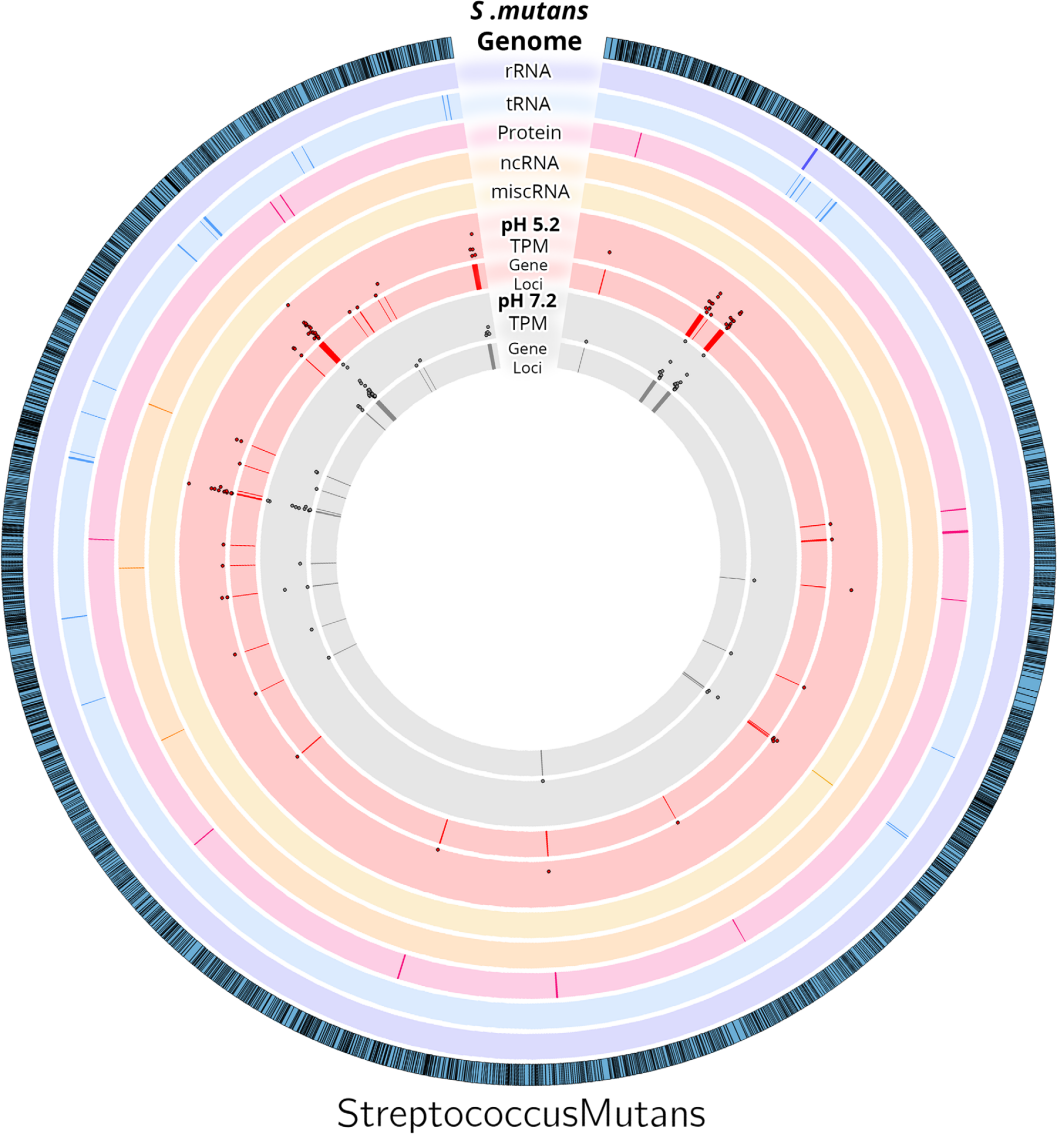
Spatial location of RNA isolated from *S. mutans* MVs. The Circos graph provides a comprehensive visualization of the *Streptococcus mutans* genome and associated RNA sequencing data. (a) The outermost blue ring represents the entire *S. mutans* genome. (b) The subsequent five tracks depict RNA‐sequencing data, color‐coded as follows: purple for rRNA, blue for tRNA, pink for protein‐coding regions, orange for ncRNA, and gold for miscellaneous RNA (miscRNA). (c) The innermost four tracks illustrate RNA packaged in membrane vesicles, with red representing MVs grown at pH 5.2 and gray representing MVs grown at pH 7.2. The scatter plots within these tracks display transcript abundance as transcripts per million (TPM), providing a comparative view of RNA packaging at different pH conditions.

MV RNA‐seq data analysis was performed with the *S. mutans* genome (Figure 6). This visualization highlights the distribution and abundance of RNA classes across the genome and demonstrates how environmental pH influences RNA packaging in MVs. The observed shifts in RNA composition and packaging profiles may have important implications for the biological functions of MVs and their role in stress response and pathogenicity, reinforcing the idea that RNA packaging is highly selective and pH dependent, which could contribute to *S. mutans* survival and virulence under varying environmental conditions. Through this analysis, we identified clusters of tRNA and protein‐coding transcripts that were packaged in the MVs produced at pH 5.2, which were absent at pH 7.2. This could suggest that specific regions are targeted under acidic conditions.

## 4 Discussion

Our study provides an analysis of *S. mutans* MVs under varying pH conditions, highlighting differences in morphology, molecular content, and RNA composition. These findings contribute to a deeper understanding of MV composition and potential functional roles in microbial adaptation and pathogenicity. Our observation that MVs derived from acidic conditions are smaller and more abundant is consistent with previous work demonstrating increased MV release and reduced vesicle size under acidic pH (Cao et al. 2020). However, by integrating RNA‐seq of vesicle‐associated RNA as well as comparing *S. mutans* whole‐cell RNA under the same conditions, our study extends these findings to show that environmental pH is also associated with marked shifts in MV RNA cargo.

Through NTA and electron microscopy, we observed that MVs derived from *S. mutans* exhibited significant variability in size and concentration across different pH conditions. MVs isolated from acidic environments were generally smaller and more abundant than those from neutral conditions. These differences suggest that pH may influence MV biogenesis, possibly by modulating membrane stress responses and vesicle formation mechanisms. These changes may affect the stability of MVs and their internal cargo.

To determine these changes, proteomic and transcriptomic analyses revealed distinct molecular signatures associated with MVs from different pH conditions. Protein content analysis from SDS gel revealed differences in protein content in MVs derived from acidic conditions, while neutral pH‐derived MVs exhibited a broader range of proteins. RNA content analysis confirmed the presence of both coding and noncoding RNAs, reinforcing the idea that MVs carry diverse RNAs involved in intercellular communication and MV RNA composition. Comparative transcriptomic analysis demonstrated significant shifts in RNA composition associated with MV‐derived RNA. These findings suggest that MVs may serve as vehicles for environmental adaptation by selectively packaging and transferring stress‐related transcripts.

Examination of rRNA packaging patterns indicated a selective enrichment of 5S rRNA fragments in MVs, potentially reflecting a role in ribosome assembly or translational regulation. Although much remains unknown, 5S rRNA is believed to mediate allosteric interactions between ribosomal functional centers, playing a key role in ribosome assembly (Stepanov and Fox 2021). The 5S rRNA interacts with various structural elements connected to the peptidyl transferase center, elongation factor binding site, and mRNA decoding region. Because of its crucial role in ribosome function, it has remained highly conserved throughout evolution (Stepanov and Fox 2021). Notably, 5S rRNA is an integral component of the large ribosomal subunit in all known organisms except mitochondrial ribosomes in fungi and animals (Szymanski et al. 2002). Studies suggest that it enhances protein synthesis by stabilizing ribosome structure and is a key catalytic component of the peptidyl transferase system (Szymanski et al. 2002). This shift may suggest alterations in MV stability under stressful conditions or an adaptive response involving selective cargo packaging.

Additionally, tRNA profiling revealed a difference in specific tRNA species, which suggests selective packaging mechanisms that may influence protein synthesis under different environmental conditions. The tRNAs that were more abundant in the acidic environment may contribute to acid tolerance by fueling proton‐scavenging pathways, ammonia production, and cell envelope modification that may help maintain pH homeostasis (Baker et al. 2017; Gillner et al. 2013). Other tRNAs may be more abundant in the MVs because they are involved in nonessential functions, allowing the cell to conserve energy and prioritize acid tolerance mechanisms (Wenderska et al. 2017). Diallo et al. tracked Ile tRFs in *E. coli* outer MVs during thermal and nutritional stress and observed that these Ile tRFs could be transferred to human cells, promoting immune responses and MAP3K4 expression (Diallo et al. 2022). Another study suggests that bacterial MVs, following endocytosis, can activate innate immune receptors by releasing their RNA cargo (Tsatsaronis et al. 2018). The RNA can then engage with receptors like TLR3, TLR7, and TLR8, leading to activation of further inflammatory mediators (Tsatsaronis et al. 2018). These trends indicate that MV cargo composition may not be random but rather a regulated process that reflects the adaptive needs of *S. mutans* in response to environmental challenges.

Using whole‐cell *S. mutans* RNA‐seq data, pathway enrichment analysis revealed several pathways that were significantly upregulated under acidic growth conditions, indicating broad shifts in cellular physiology. Among these, l‐histidine biosynthesis was enriched at pH 5.2. Histidine and histidine‐derived signaling systems, including histidine kinases, play a key role in bacterial signal transduction and adaptation to environmental stress, suggesting that increased histidine biosynthesis may support stress‐responsive regulatory processes under acidic conditions (Dutta et al. 1999). Pathways involved in ATP biosynthesis and the generation of precursor metabolites and energy were also enriched. This enrichment is consistent with increased energetic demands associated with growth and stress adaptation at low pH and may provide the metabolic capacity required to support cellular processes that are enhanced under acidic conditions, including increased MV production (Baeza and Mercade 2021; L. Chen and Tai 1985). Additionally, fatty acid and lipid biosynthesis pathways were enriched at pH 5.2. Given that MVs are composed largely of lipids and fatty acids, upregulation of these pathways is consistent with altered MV production under acidic stress (Toyofuku et al. 2023; Xu et al. 2023). Together, these enriched pathways provide context for observed changes in MV abundance, size, and cargo composition under acidic conditions.

Several pathways were significantly depleted in whole‐cell *S. mutans* grown at pH 5.2, including the tRNA charging and aminoacyl‐tRNA charging pathways. tRNA charging is directly linked to bacterial growth and accumulation rates, as it regulates both translational capacity and the balance between charged and uncharged tRNAs (Toyofuku et al. 2023). Depletion of this pathway under acidic conditions is consistent with activation of stress‐associated growth limitation, which aligns with the reduced growth rate observed in our growth curve analysis. Reduced tRNA charging may change how the cell makes proteins during stress, helping the bacteria focus on producing proteins needed for survival while reducing the production of proteins that are not essential under stressful conditions (Fruchard et al. 2025; Parker et al. 2020). The macromolecule modification pathway was also depleted at pH 5.2, which is consistent with bacteria reducing energy‐intensive processes such as ribosome modification and protein processing during stress to conserve resources (Njenga et al. 2023). Collectively, the depletion of these pathways indicated that *S. mutans* prioritizes stress adaptation, which may contribute to the altered RNA cargo within the MVs observed in this study.

Incorporating whole‐cell RNA‐seq alongside MV RNA‐seq was essential for interpreting MV cargo composition within its cellular context. Analyzing whole‐cell RNA under identical growth conditions allowed us to distinguish RNA changes that reflect overall cellular responses from those that are differentially represented within MVs. Comparison of whole‐cell and MV‐associated RNA profiles revealed opposing trends in several RNA classes and individual transcripts, indicating that MV RNA content does not simply mirror cellular RNA abundance. Furthermore, pathway enrichment analysis of whole‐cell transcriptomes provided insight into stress‐associated shifts in metabolism, translation, and membrane remodeling that likely influence MV production and cargo composition. Together, inclusion of whole‐cell transcriptomic data helped clarify MV RNA content and showed that MV composition reflects the cell's response to stress, rather than being simply carried over from the cell.

Our findings suggest that MV biogenesis and cargo composition are pH dependent, supporting the idea of selective packaging under environmental stress. Studies have demonstrated that bacteria under stress may either increase MV production, enhance the export of damaged or immature cargo into MVs, or both (Orench‐Rivera and Kuehn 2016). Additionally, multiple bacteria have been shown to adapt to a change of environment, where they have shown changes in MV contents. For example, in an iron‐deficient environment, *Haemophilus influenzae* and *Vibrio cholerae* show an increase in phospholipids and certain fatty acids, and *Vibrio fischeri* upregulates its outer membrane protein OmpU under acidic conditions (Xiu et al. 2024). This supports the idea that MVs serve as a protective mechanism, aiding in bacterial survival through dispersal, nutrient trafficking, horizontal gene transfer, phage decoying, and antimicrobial resistance (Mozaheb and Mingeot‐Leclercq 2020).

These vesicles can communicate with other microbes and host cells through molecules they carry, such as DNA and RNA, and can influence signaling cascades by triggering Toll‐like receptors (TLRs) through their proteins, lipids, and nucleic acids (Villageliu and Samuelson 2022; Xiu et al. 2024). Studies have shown that MVs can transport RNA to host cells, where they may regulate or adjust host gene expression (Xiu et al. 2024). Because of this influence, MVs may affect endocrine and immune pathways, which in turn affect metabolism and inflammatory responses, suggesting potential roles in diseases such as type II diabetes (Villageliu and Samuelson 2022). Bacterial MVs contribute to disease mechanisms and progression by delivering virulent cargo or creating biofilms (Villageliu and Samuelson 2022). Studies have also shown that when MVs collected from mice with heatstroke are injected into healthy mice, multiple organs exhibit damage, inflammatory cell infiltration, and elevated inflammatory cytokine levels (Li et al. 2022; Xiu et al. 2024). This is important because if MVs contribute to disease and inflammatory processes, they could represent a potential target for therapeutics.

Understanding the molecular mechanisms of MV formation and cargo selection has important implications for the understanding of bacterial pathogenesis and for therapeutic targeting. Given the role MVs play in multiple diseases, disrupting MV production or altering specific mechanisms could serve as a means to mitigate disease severity. MVs play key roles in conditions such as airway inflammation (Ryu et al. 2023), fetal complications and preterm birth (Surve et al. 2016), systemic inflammatory response syndrome (SIRS) (Tian et al. 2023), sepsis (Tian et al. 2023), atopic dermatitis (Harvey‐Seutcheu et al. 2024), periodontal disease (S. Chen et al. 2023), pulmonary inflammation (S. Chen et al. 2023), gastrointestinal disorders (S. Chen et al. 2023), atherosclerosis (S. Chen et al. 2023), and Alzheimer's disease (S. Chen et al. 2023).

Understanding how *S. mutans* responds to acidic conditions is essential for oral health, particularly in the context of the Stephan curve, which describes pH changes in the oral cavity following sugar consumption. The curve consists of three phases: an initial rapid pH drop due to bacterial fermentation of sucrose, enamel demineralization if the pH falls below 5.5, and a gradual return to baseline within 30–60 min as saliva neutralizes the acid. Since this study examines *S. mutans* MVs at pH 5.2, a level associated with enamel demineralization, these findings offer insight into how *S. mutans* adapts to cariogenic conditions, potentially influencing biofilm formation and disease progression (Catunda et al. 2023). While this study provides valuable insights, it is limited by examining only two pH conditions, which may not fully capture the entirety of environmental influences on MV composition. Additionally, *S. mutans* was the only bacteria studied; additional bacterial species or complete oral microbiomes need to be evaluated in order to fully understand the development of bacterial MVs in a complex system.

Overall, this study highlights an aspect of *S. mutans* physiology, demonstrating how environmental conditions influence MV composition. These findings advance our understanding of bacterial adaptations and communication, with potential implications for targeting pathogens.

## Funding

The authors have nothing to report.

## Conflicts of Interest

The authors declare no conflicts of interest.

## Supporting information




**Supplemental Figure 1. Growth Curve of *S. mutans* in pH 5.2 and pH 7.2 environments**. The chart illustrates the growth curves of *S. mutans* under different conditions over 42 hours, with OD_600_ readings taken every 30 minutes at 37°C. It compares growth rates across various dilution levels in pH 7.2 and pH 5.2 environments. In pH 7.2, bacterial growth increased rapidly before plateauing around 5.00 × 10^7^ CFU/mL, whereas in pH 5.2, growth stabilized at approximately 2.57 × 10^7^ CFU/mL.

**Supplemental Figure 2. Altered protein compositions in MVs based on pH**. Comparison of protein content in MVs grown at pH 7.2 and pH 5.2, highlighting differences in protein levels between the two conditions. Protein profiles were analyzed using SDS‐PAGE, revealing variations in band patterns and intensities. Notably, MVs grown at pH 7.2 exhibited distinct bands at approximately 160 kDa, 67 kDa, and 61 kDa, indicating potential differences in protein composition

**Supplemental Figure 3. Individual analysis of tRNAs in MVs**. Different types of tRNAs identified within *S. mutans* MVs (A) and within the small RNA *S. mutans* whole cells (B), with significant differences indicated by * (*p* < 0.05), ** (*p* < 0.01), or **** (*p* < 0.0001).

## Data Availability

The data that support the findings of this study are openly available in NCBI SRA at https://www.ncbi.nlm.nih.gov/sra, reference number PRJNA1413374.
