# Supplementary figures and images for: Environmental Stress Induces Altered Composition of Streptococcus mutans Membrane Vesicles: pH‐Driven Changes in Membrane Vesicle Production and Composition
Source: Mol Oral Microbiol. 2026 Feb 20;41(3):145–57. doi: 10.1111/omi.70022 (PMC13121933; doi:10.1111/omi.70022)

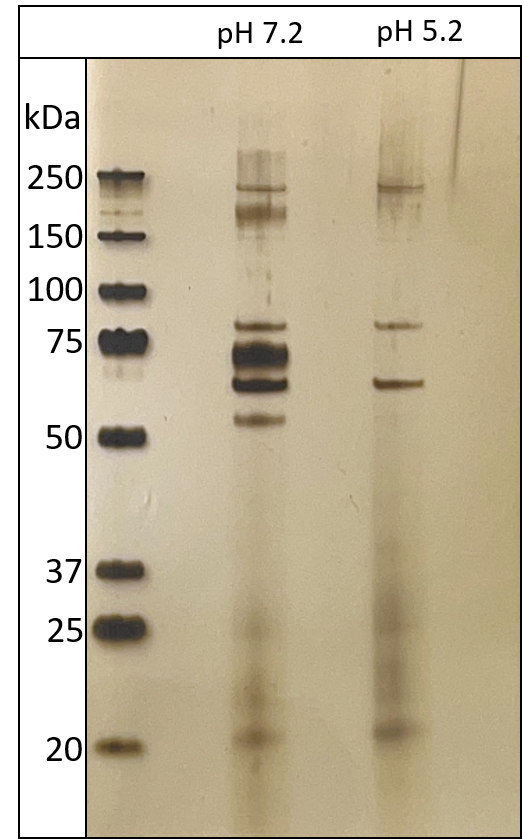

Supplement: Supplementary file 2 — Supplemental Figure 2. Altered protein compositions in MVs based on pH. Comparison of protein content in MVs grown at pH 7.2 and pH 5.2, highlighting differences in protein levels between the two conditions. Protein profiles were analyzed using SDS‐PAGE, revealing variations in band patterns and intensities. Notably, MVs grown at pH 7.2 exhibited distinct bands at approximately 160 kDa, 67 kDa, and 61 kDa, indicating potential differences in protein composition [file OMI-41-145-s002.tif]
